# Genome-wide enriched pathway analysis of acute post-radiotherapy pain in breast cancer patients: a prospective cohort study

**DOI:** 10.1186/s40246-019-0212-8

**Published:** 2019-06-13

**Authors:** Eunkyung Lee, Cristiane Takita, Jean L. Wright, Susan H. Slifer, Eden R. Martin, James J. Urbanic, Carl D. Langefeld, Glenn J. Lesser, Edward G. Shaw, Jennifer J. Hu

**Affiliations:** 1 Department of Health Sciences, University of Central Florida College of Health Professions and Sciences, Orlando, FL 32816 USA; 2 Department of Public Health Sciences, University of Miami Miller School of Medicine, Miami, FL 33136 USA; 3 Sylvester Comprehensive Cancer Center, University of Miami Miller School of Medicine, Miami, FL 33136 USA; 4 Department of Radiation Oncology, University of Miami Miller School of Medicine, Miami, FL 33136 USA; 5 Department of Radiation Oncology and Molecular Radiation Sciences, Johns Hopkins University, Baltimore, MD 21231 USA; 6 The Center for Genetic Epidemiology and Statistical Genetics, Joph P. Hussman Institute for Human Genomics, University of Miami Miller School of Medicine, Miami, FL 33136 USA; 7 Wake Forest NCORP Research Base, Winston-Salem, NC 27157 USA

**Keywords:** Breast cancer, Radiotherapy, Pain, Pathway analysis, Genetic variants

## Abstract

**Background:**

Adjuvant radiotherapy (RT) can increase the risk of developing pain; however, the molecular mechanisms of RT-related pain remain unclear. The current study aimed to identify susceptibility loci and enriched pathways for clinically relevant acute post-RT pain, defined as having moderate to severe pain (pain score ≥ 4) at the completion of RT.

**Methods:**

We conducted a genome-wide association study (GWAS) with 1,344,832 single-nucleotide polymorphisms (SNPs), a gene-based analysis using PLINK set-based tests of 19,621 genes, and a functional enrichment analysis of a gene list of 875 genes with *p* < 0.05 using NIH DAVID functional annotation module with KEGG pathways and GO terms (*n* = 380) among 1112 breast cancer patients.

**Results:**

About 29% of patients reported acute post-RT pain. None of SNPs nor genes reached genome-wide significant level. Four SNPs showed suggestive associations with post-RT pain; rs16970540 in *RFFL* or near the *LIG3* gene (*p* = 1.7 × 10^−6^), rs4584690, and rs7335912 in *ABCC4/MPR4* gene (*p* = 5.5 × 10^−6^ and *p* = 7.8 × 10^−6^, respectively), and rs73633565 in *EGFL6* gene (*p* = 8.1 × 10^−6^). Gene-based analysis suggested the potential involvement of neurotransmitters, olfactory receptors, and cytochrome P450 in post-RT pain, whereas functional analysis showed glucuronidation (FDR-adjusted *p* value = 9.46 × 10^−7^) and olfactory receptor activities (FDR-adjusted *p* value = 0.032) as the most significantly enriched biological features.

**Conclusions:**

This is the first GWAS suggesting that post-RT pain is a complex polygenic trait influenced by many biological processes and functions such as glucuronidation and olfactory receptor activities. If validated in larger populations, the results can provide biological targets for pain management to improve cancer patients’ quality of life. Additionally, these genes can be further tested as predictive biomarkers for personalized pain management.

**Electronic supplementary material:**

The online version of this article (10.1186/s40246-019-0212-8) contains supplementary material, which is available to authorized users.

## Background

Breast cancer is the most frequently diagnosed cancer and the second leading cause of cancer death in American women [1]. Early detection and improved treatment modalities have led to a remarkable reduction in the mortality rate of breast cancer patients, and currently more than 3.5 million breast cancer survivors are living in the USA [2]. Given that approximately 70% of breast cancer patients receive adjuvant radiotherapy (RT) after breast surgery to improve clinical outcomes [3], it is critical to address cancer survivorship issues relating to RT-induced symptoms which may affect the quality of life (QOL). Among many symptoms, pain occurs in up to 60% of breast cancer survivors [4, 5], where more than half of them report moderate to severe pain [4]. Unmanaged pain can interrupt planned RT schedules and impact the accurate delivery of therapeutic radiation doses to tumor tissues, which can thus diminish the potential benefits of adjuvant RT. Persistent pain after cancer treatment is also critical, affecting cancer survivor’s functional performance and productivity. Moreover, once pain develops, it may last for more than 17 years after completion of RT [6].

In addition to RT planning and treatment parameters, age, body mass index (BMI), medication, lifestyle factors such as smoking and exercise, and coexisting morbidities can contribute to pain perception during RT [7, 8]; however, inter-individual genetic variations can also influence post-RT pain severity. Several studies have reported genetic variants associated with cancer treatment-related pain among breast cancer patients. For example, genotype AA for interleukin (IL)-13 single-nucleotide polymorphism (SNP) rs1295686 was associated with both pain and lymphedema after breast cancer surgery [9]. Also, SNPs in cytokine genes *IFNG1*, *IL*, and *NFKB1* have been associated with severe breast pain following breast cancer surgery [10]. Genetic variations in cytidine deaminase (*CDD*) contributed to chemotherapy-induced neuropathy [11]. Furthermore, variations in cytochrome P450 (*CYP*) and vitamin D receptor (*VDR*) genes have been associated with aromatase inhibitor-related arthralgia [12]. However, there is a scientific knowledge gap regarding the molecular mechanisms or the genetic variants influencing pain in patients receiving adjuvant RT.

Thus, to identify susceptibility loci for post-RT pain, we completed a genome-wide association study (GWAS) of 1,344,832 SNPs in a prospectively followed cohort of breast cancer patients undergoing adjuvant RT for breast cancer. As part of this study, we completed gene-based association analyses and functional enrichment pathway analyses to describe the biological profiles underlying genetic mechanisms of post-RT pain. Gene-based association approach considers the joint actions of multiple SNPs within a gene and assigns a representative *p* value for a gene. If a gene contains more than one causative SNPs with small or moderate effect, then joint effects of several SNPs within that gene may be more detectable than single SNP effect. Functional enrichment pathway analysis, using the gene list produced by gene-based association analyses, is complementary to GWAS in finding risk loci as well as interpreting GWAS results in terms of biological features or function.

## Materials and methods

### Study populations

This study analyzed 1112 participants from two cohort studies which employed the same protocol to evaluate the impact of molecular genomics on radiosensitivity among breast cancer patients. The first study population consisted of a cohort of 513 women with newly diagnosed, histologically confirmed breast cancer, recruited from the Department of Radiation Oncology of the University of Miami (UM) Sylvester Comprehensive Cancer Center, University of Miami Hospital, and Jackson Memorial Hospital between December 2008 and January 2014. We obtained sufficient quantity and quality of DNA for 458 patients, and among these, 377 patients with complete genotype and pain data were included in the current study. The second study population consisted of a nationwide cohort of breast cancer patients who were enrolled in the Wake Forest (WF) National Cancer Institute Community Clinical Oncology Program (CCOP) Research Base 97609 Study. This study enrolled 1000 patients between November 2011 and August 2013. Among these, 728 patients with complete genotype and pain data were included in the current analysis. Protocols were approved by each participating site’s Institutional Review Boards, and written informed consent was obtained from each study participant before entering the study.

Each patient completed a baseline questionnaire and provided blood samples (20 ml) before the initiation of RT (baseline) and immediately after completion of RT (post-RT). Blood samples from participants enrolled in the WF Research Base 97609 study were transported to the University of Miami via overnight shipping for DNA extraction and genotyping. All the DNA samples were stored at − 20 °C until assay.

### Radiation treatment

Detailed information on radiation treatment was described in the previous papers [13, 14]. In brief, RT was delivered using 6 or 10 MV standard or partially wide photon tangents with a forward planned field-in-field technique to maximize dose homogeneity. In general, patients received a total dose of 42.4 to 66 Gy to their intact breast or chest wall for 3 to 7 weeks depending on both the fractionation scheme and additional boost.

### Phenotype definition: post-RT pain

All women enrolled in the study filled out the National Surgical Adjuvant Breast and Bowel Project (NSABP) B-39/Radiation Therapy Oncology Group (RTOG) 0413 protocol QOL questionnaire at baseline and post-RT, which contains four pain severity items (i.e., pain at its worst, least, average during the past 4 weeks, and now) from the Brief Pain Inventory (BPI). A pain score was determined as the mean of these four pain severity items (from 0 = no pain to 10 = the worst imaginable pain) as suggested by the BPI developers [15], and moderate to severe pain (pain score ≥ 4) was considered clinically relevant [16, 17]. Therefore, cases were defined as those that had a pain score ≥ 4 at post-RT (*n* = 326), and the reference group included those with a pain score < 4 at post-RT (*n* = 786).

### Genotyping and quality control

Genomic DNA was extracted from frozen whole blood using the QIAamp DNA Blood Mini kit (Qiagen, Inc., Valencia, CA), and the DNA genotype was screened for ∼ 2,500,000 haplotype tagging SNPs using an Illumina HumanOmni2.5-8v1 BeadChip (Illumina, San Diego, CA) according to Illumina protocols at the University of Miami Hussman Institute for Human Genomics Genotyping Core. Both genotype clustering and calling were performed using Illumina’s GenomeStudio V2011.1 software. The genotyping quality control/assurance included (i) four internal controls in each plate, (ii) randomly assigned case and reference samples in each plate to avoid any biases between plates, and (iii) the Hardy-Weinberg equilibrium (HWE) test to identify problematic SNPs. SNPs were excluded from the analysis if they had no genotype for > 5% of individuals, were not in HWE within a reference group (using threshold *p* < 1.0 × 10^−6^) or had minor allele frequency < 5%. Subjects were also excluded if they had > 5% of all variants missing. The final dataset contained 1,344,832 SNPs with a genotype call rate of 99.8%. All the quality control procedures were conducted using PLINK (v1.09) (http://zzz.bwh.harvard.edu/plink/) [18].

### Population substructure

Population substructure was evaluated using principal component analysis (PCA). To remove outliers, we first computed the analysis with a randomly selected and pruned subset of 30,929 common SNPs (LD = 0.5 and minor allele frequency = 0.05) for the study subjects as well as four reference populations from the International HapMap/1000Genomes Project: 85 European-Americans from Utah (CEU); 88 Yorubans from Ibadan, Nigeria (YRI); 97 Han Chinese from Beijing, China (CHB); and 89 Japanese in Tokyo (JPT). Next, we computed the analysis for the study subjects only without the reference populations merged in to determine principal components (PCs) for covariates. The first three PCs were included to adjust for population substructure to minimize spurious associations and test inflation and improve power to detect true associations in subsequent analyses. PCA was performed using EIGENSTRAT v5.0 (https://reich.hms.harvard.edu/software) [19].

### Statistical analysis

#### Single marker genome-wide association analyses

Pearson’s chi-square test or Fisher’s exact test were used to find the potential risk factors for post-RT pain, which compared proportions of patients with post-RT pain by study variables in univariate analysis. These factors were further included in the multivariable logistic regression analysis. The variables that were identified as significant in multivariable analysis were then included in subsequent analyses to adjust for potential confounding effects: surgery type (mastectomy vs lumpectomy), age (continuous), BMI (continuous), smoking (never vs. ever), the number of comorbidities (0, 1, vs 2+), pre-RT pain score (< 4 vs. ≥ 4), and population sub-stratification (PC1, PC2, PC3).

The associations between post-RT pain and genotype frequency, assuming an additive genetic model for minor allele counts of SNPs coded as 0/1/2, were assessed using multivariable logistic regression after adjusting for aforementioned potential confounders. The odds ratios (ORs) and 95% confidence intervals (95% CIs) for each SNP are reported. A quantile-quantile (Q-Q) plot of observed versus expected chi-square test statistics and estimated inflation factor confirmed the tests met the distributional assumptions. The genome-wide significance was set at the standard *p* < 5 × 10^−8^ to account for the number of tests. General data management and statistical analyses were performed using PLINK and R (http://cran.r-project.org/). A Manhattan plot for the result was generated using R package, qqman.

We estimated the statistical power using the software program, PS Power, and Sample Size Program [20]. Given 326 cases and 786 controls with minor allele frequency = 0.24 and alpha = 5 × 10^−8^, we had 80% power to detect an OR of 2.41 for an association between a SNP and post-RT pain.

#### Gene-based association analysis

First, a total of 950,621 SNPs were mapped to 19,621 genes according to genomic positions on the Ensembl/Entrez hg19/GRCh37 Consensus Genes, which were downloaded on 3 September 2016 from the Figshare, the online academic digital repository (https://figshare.com/articles/hg19_GRCh37_Consensus_Genes/103113/4) [21] using ± 20 kb gene boundaries as delimiters to include regulatory SNPs [22]. These genes are consistently annotated across Ensembl and Entrez-gene databases and have HUGO gene symbol identifiers.

Second, gene-based association analyses were performed using PLINK set-based tests, which required raw genotype data as input and aggregate *p* values from the set of SNPs within a gene accounting for linkage disequilibrium (LD) and gene size with phenotype permutation. Although its computational burden is high, PLINK set-based tests are more relevant in the current study where we are more interested in joint effects of multiple SNPs with moderate effects. PLINK performs a single SNP association analysis for each gene accounting for the covariates. A mean SNP statistic is calculated from the significant and independent set of SNPs under the defined *p* value and LD threshold setting. The empirical *p* value for the gene is calculated after repeated analysis in simulated datasets with permutation of the phenotype. The empirical *p* value indicates the number of times the test statistics of the simulated gene exceed that of the original gene. Gene with empirical *p* value < 2.5 × 10^−6^, a Bonferroni-corrected threshold (≈ 0.05/19,621), was considered significant accounting for multiple testing corrections. The parameters in PLINK set-based test for the current study were set at *p* (*p* value threshold for selection of SNPs from a single SNP association) < 0.05, LD r2 (pair-wise correlation between two SNPs) < 0.5, mperm (number of permutation) = 10,000, and set-max (max number of SNPs in a gene) = 99,999.

#### Pathway analysis

To identify which biological terms/functions are specifically enriched with post-RT pain, we conducted pathway analysis of the GWAS results. The Kyoto Encyclopedia of Genes and Genomes (KEGG) and Gene Ontology (GO) terms were used for functional annotation and enrichment analyses. In total, 530 pathways with minimum gene size ≥ 5 were analyzed since small pathways can exhibit spurious associations due to large single locus effects [23]. A total of 875 genes having *p* < 0.05 in PLINK gene-based association analyses were selected for pathway analysis. Modified Fisher’s exact tests were performed using the web-based gene-enrichment analysis tool, the Database for Annotation, Visualization and Integrated Discovery (DAVID, https://david.ncifcrf.gov/) v6.8 [24], and a pathway with the false discovery rate (FDR) < 0.05 after accounting for multiple testing was considered significant.

## Results

### Patient characteristics and post-RT pain

The study population across two datasets consisted of 401 Hispanic Whites (HW, 36%), 357 non-Hispanic Whites (NHW, 32%), 296 black or African Americans (AA, 27%), and 58 of other races (5%). Mean (±SD) age at the time of enrollment was 57.4 ± 10.5 years (range 23.5 – 88.9) and 77% of patients were overweight or obese. 86% of patients received post-lumpectomy RT, and 14% had post-mastectomy RT. They were treated with a mean of 58.6 ± 5.7 Gy radiation dose to either the whole breast or the chest wall.

A total of 326 (29%) patients showed clinically relevant post-RT pain. Patient-, tumor-, and treatment-related factors that may be related to post-RT pain were compared between case and reference groups (Table 1). Those who were AA or HW women, younger, obese, ever smoked, had comorbidities ≥ 2, had received mastectomy, conventionally fractionated RT, and whose pre-RT pain score ≥ 4 were more likely to report post-RT pain.

**Table 1:** Characteristics of study populations by post-RT pain

Variable	Categories			Post-RT pain					
		Total		No (score < 4)		Yes (score ≥ 4)			
		*N*	%	*N*	%	*N*	%	*p* ^1^	*p* ^2^
Study population		1112	100	786	71	326	29		
Study site	UM	377	34	243	64	134	36	*0.001*	0.340
	WFU	735	66	543	74	192	26		
Race/ethnicity	AA	296	27	194	66	102	34	*< 0.001*	0.315
	HW	401	36	265	66	136	34		
	NHW	357	32	282	79	75	21		
	Other	58	5	45	78	13	22		
Age at consent (years)	< 50	283	25	175	62	108	38	*<.0001*	*<.0001*
	50–59	372	34	247	66	125	34		
	≥ 60	457	41	364	80	93	20		
BMI (kg/m^2^)	< 25	257	23	208	81	49	19	*< .0001*	*0.002*
	25–29.99	357	32	263	74	94	26		
	≥ 30	498	45	315	63	183	37		
Smoking history	Never	706	64	514	73	192	27	*0.033*	*0.026*
	Ever	390	35	260	67	130	33		
No. of comorbidity^3^	0	464	42	337	73	127	27	*0.035*	*0.001*
	1	378	34	275	73	103	27		
	≥ 2	270	24	174	64	96	36		
Tumor stage	0	217	20	159	73	58	27	0.077	–
	I	528	47	384	73	144	27		
	II	234	21	161	69	73	31		
	III–IV	132	12	82	62	50	38		
ER	Positive	894	80	632	71	262	29	0.957	–
	Negative	217	20	153	71	64	29		
PR	Positive	767	69	533	69	234	31	0.178	–
	Negative	343	31	252	73	91	27		
HER2	Positive	143	13	106	74	37	26	0.283	–
	Negative	798	72	556	70	242	30		
Triple negative	No	964	87	687	71	277	29	0.248	–
	Yes	134	12	89	66	45	34		
Surgery	Lumpectomy	959	86	698	73	261	27	*< .0.001*	*0.023*
	Mastectomy	153	14	88	58	65	42		
Chemotherapy	No	610	55	441	72	169	28	0.193	**–**
	Yes	502	45	345	69	157	31		
Hormonal therapy	No	785	71	570	73	215	27	*0.029*	0.587
	Yes	327	29	216	66	111	34		
RT fractionation	Conventional	972	87	670	69	302	31	*0.003*	0.414
	Hypo	138	13	114	83	24	17		
	Partial	2	0.1	2	100	.	.		
RT dose (Gy)	< 60	266	24	205	77	61	23	*0.010*	–
	≥ 60	834	75	574	69	260	31		
Boost	No	121	11	94	78	27	22	0.078	–
	Yes	979	88	685	70	294	30		
Pre-RT pain score	< 4	936	84	715	76	221	24	*< .0001*	*< .0001*
	≥ 4	151	14	57	38	94	62		

^1^*p* values from chi-square or Fisher’s exact test. Significant findings are in italics

^2^*p* values from multivariable logistic regression adjusting for other variables in tables

^3^The number of comorbidities, sum of 12 patient-reported comorbid conditions: diabetes, hypertension, heart disease, lung disease, thyroid condition, cirrhosis liver, stroke, chronic bronchitis, hepatitis, tuberculosis, and 2 others. *AA* African American; *HW* Hispanic Whites; *NHW* = non-Hispanic Whites; *BMI* body mass index

### Genome-wide single-marker association analyses

**Fig. 1:**
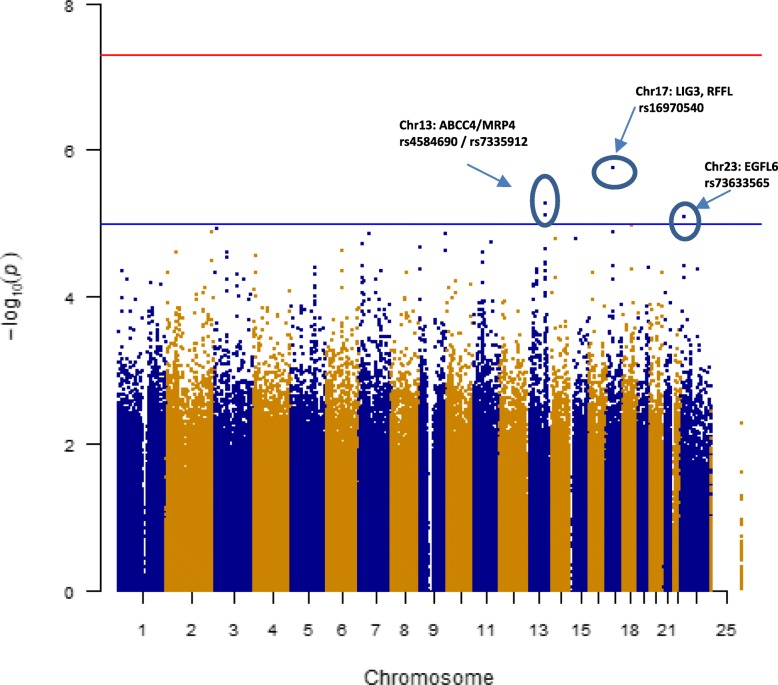
Manhattan plot for post-RT pain among breast cancer patients. This figure shows the *p* values of the SNPs after applying the additive genetic model in the multivariable logistic regression model by genomic location. No region exceeded genome-wide significance in the sample. Red line indicates genome-wide significance level of 5 × 10^−8^ and blue line indicates suggestive level of significance of 1 × 10^−5^

Genome-wide single SNP associations were conducted with 1,344,832 SNPs that passed quality control. The Q-Q plot (Additional File 1: Fig. S1) showed no evidence for test statistic inflation due to population substructure (inflation factor 1.016). None of SNPs achieved a genome-wide significance level of *p* < 5 × 10^−8^ (Fig. 1). Four SNPs showed associations with post-RT pain at the marginal significance level of *p* < 1 × 10^−5^; rs16970540 in ring finger and FYVE-like domain containing E3 ubiquitin protein ligase (*RFFL)* or near to DNA ligase 3 (*LIG3*) gene (*p* = 1.7 × 10^−6^), rs4584690, and rs7335912 in ATP-binding cassette, sub-family A, member 4 (*ABCC4)/*multidrug resistance protein 4 *(MRP4)* gene (*p* = 5.5 × 10^−6^ and *p* = 7.8 × 10^−6^, respectively), and rs73633565 in epidermal growth factor-like protein 6 (*EGFL6*) gene (*p* = 8.1 × 10^−6^). The top 30 significant SNPs are summarized in Table 2. For rs16970540, those who had at least one minor T allele were 2.2 times more likely to have post-RT pain compared to those who had C allele (95% CI = 1.59 – 3.04).

**Table 2:** Top 30 SNPs from genome-wide association study of post-RT pain among breast cancer patients

			Allele							
Chr	SNP	BP	Minor	Major	MAF	OR^1^	95% CI		*P*value^1^	Nearest gene
17	rs16970540	33,338,447	T	C	0.10	2.20	1.59	3.04	1.73E-06	RFFL, LIG3
13	rs4584690	95,680,132	T	C	0.18	1.85	1.42	2.42	5.46E-06	ABCC4(MRP4)
13	rs7335912	95,667,680	G	A	0.19	1.80	1.39	2.33	7.81E-06	ABCC4(MRP4)
23	rs73633565	13,477,311	G	A	0.16	1.90	1.43	2.52	8.06E-06	EGFL6
18	rs986117	44,128,503	C	A	0.36	1.62	1.31	2.01	1.04E-05	LOXHD1
3	rs6800849	11,790,188	A	C	0.17	1.84	1.40	2.41	1.15E-05	VGLL4
2	rs13031207	226,076,914	G	A	0.23	1.72	1.35	2.20	1.30E-05	DOCK10
17	rs10083888	33,402,980	T	C	0.11	2.00	1.47	2.73	1.30E-05	RFFL
7	rs1108228	51,787,366	A	G	0.46	1.61	1.30	1.99	1.40E-05	COBL
9	rs10901329	133,924,008	G	A	0.43	0.61	0.49	0.76	1.41E-05	LAMC3
15	rs4497630	30,276,341	A	G	0.26	0.57	0.45	0.74	1.62E-05	FAM7A3
14	rs11849204	34,332,971	G	A	0.11	1.98	1.45	2.70	1.62E-05	NPAS3
11	rs76647546	88,929,531	G	A	0.06	2.57	1.67	3.97	1.84E-05	TYR
7	rs12374901	15,024,954	T	C	0.43	0.62	0.50	0.77	1.88E-05	DGKB
9	rs10973146	3,706,109	G	A	0.06	2.42	1.61	3.65	2.13E-05	GLIS3
13	rs17189292	95,685,572	G	A	0.13	1.96	1.44	2.68	2.21E-05	ABCC4(MRP4)
6	rs72904381	83,130,109	C	T	0.45	0.63	0.51	0.78	2.30E-05	TPBG
9	rs2275139	133,924,072	C	T	0.43	0.62	0.50	0.78	2.35E-05	LAMC3
11	rs10901897	50,199,387	C	T	0.34	0.61	0.48	0.76	2.45E-05	LOC441601
2	rs2278358^*^	43,968,167	A	G	0.12	1.90	1.412	2.569	2.49E-05	PLEKHH2
3	rs717228	60,602,895	C	T	0.17	0.52	0.38	0.70	2.55E-05	FHIT
4	rs10023531	12,357,990	A	G	0.43	1.56	1.27	1.92	2.70E-05	HS3ST1
3	rs79082706	60,614,582	G	A	0.16	0.51	0.38	0.70	2.86E-05	FHIT
2	rs1443663	226,074,083	A	C	0.25	1.65	1.30	2.10	3.25E-05	DOCK10
13	rs4148524	95,767,333	C	T	0.14	1.84	1.38	2.45	3.45E-05	ABCC4(MRP4)
11	rs10082560	50,276,354	C	G	0.35	0.61	0.48	0.77	3.49E-05	LOC441601
13	rs12853814	95,720,188	C	T	0.13	1.93	1.41	2.64	3.55E-05	ABCC4(MRP4)
17	rs7214759	35,261,346	G	C	0.20	1.68	1.31	2.15	3.71E-05	LHX1
7	rs2731551	18,277,585	T	C	0.33	1.58	1.27	1.95	3.77E-05	HDAC9
7	rs596699	18,351,391	A	C	0.34	1.57	1.26	1.94	3.82E-05	HDAC9

OR and *p* values from multivariable logistic regression analysis assuming additive genetic model

*Chr* = chromosome, *SNP* = single-nucleotide polymorphism, *BP* = base position based on hg19/GRCh37, *MAF* = minor allele frequency, *OR* = odds ratio, *CI* = confidence intervals

### Gene-level association analyses

To identify potential risk genes consisting of multiple SNPs with a modest functional effect, we performed gene-based association analyses using PLINK set-based tests, and the results are listed in Table 3. None of them reached our Bonferroni significance threshold of *p* < 2.5 × 10^−6^. However, seven genes showed suggestive evidence of association with *p* < 5.0 × 10^−4^: *EIF4G1*, *FAM131A*, *GRID2IP*, *NMUR2*, *OR10V1*, *CYP4F22*, and *LECT1*.

**Table 3:** Top 30 genes from PLINK-set based test of post-RT pain among breast cancer patients

Gene symbol	Chr	Entrez ID	Gene name	P (gene)	No. SNPs within a gene	No. SNPs with *p* < 0.05	No. SNPs with *p* < 0.05 and *R*^2^ < 0.5	Top SNP
EIF4G1	3q27.1	1981	Eukaryotic translation initiation factor 4 Gamma 1	2.00E-04	28	1	1	rs4912540
FAM131A	3q27.1	131,408	Family with sequence similarity 131 member A	2.00E-04	22	1	1	rs4912540
GRID2IP	7p22.1	392,862	Glutamate receptor, ionotropic, delta 2 (Grid2) interacting protein 1, delphilin	2.00E-04	31	1	1	rs73674133
NMUR2	5q33.1	56,923	Neuromedin U receptor 2	2.00E-04	22	1	1	rs11739168
OR10V1	11q12.1	390,201	Olfactory receptor family 10 subfamily V member 1	2.00E-04	13	2	1	rs7937162
CYP4F22	19p13.12	126,410	Cytochrome P450 family 4 subfamily F member 22	3.00E-04	54	1	1	rs73514704
LECT1	13q14.3	11,061	Leukocyte cell-derived chemotaxin 1, chondromodulin-1	4.00E-04	23	9	4	rs3759509
LDHAL6B	15q22.2	92,483	Lactate dehydrogenase A like 6B	5.00E-04	38	7	1	rs11852359
PPP2R3B	Xp22.33	28,227	Protein phosphatase 2 regulatory subunit B″beta	0.0007	38	1	1	rs28485241
PRKCDBP	11p15.4	112,464	Protein kinase C delta-binding protein	0.0007	38	1	1	rs4604857
TRIM64C	11p11.12	646,754	Tripartite motif containing 64C	0.0007	2	1	1	rs1819409
OR52N1	11p15.4	79,473	Olfactory receptor family 52 subfamily N member 1	0.0008	14	10	1	rs11607346
PDE4D	5q12.1	5144	Phosphodiesterase 4D	0.0008	719	41	18	rs1498599
PRPH2	6p21.1	5961	Peripherin 2	0.0008	28	1	1	rs200618579
RFFL	17q12	117,584	Ring finger and FYVE like domain containing E3 ubiquitin protein ligase	0.0008	27	10	4	rs16970540
OR4C12	11p11.12	283,093	Olfactory receptor family 4 subfamily C member 12	0.0010	1	1	1	rs4242812
MMADHC	2q23.2	27,249	Methylmalonic aciduria and homocystinuria, CblD type	0.0011	17	1	1	rs13027589
OR4A47	11p11.2	403,253	Olfactory receptor family 4 subfamily A member 47	0.0011	6	3	2	rs7103557
MPO	17q22	4353	Myeloperoxidase	0.0012	19	1	1	rs8178409
OSBP	20q13.33	9885	Oxysterol-binding protein	0.0012	9	1	1	rs4938923
ANAPC13	3q22.2	25,847	Anaphase promoting complex subunit 13	0.0012	18	1	1	rs75858178
CEP63	3q22.2	80,254	Centrosomal protein 63	0.0013	29	1	1	rs75858178
ROD1	9q32	9991	Regulator of differentiation 1	0.0014	33	20	2	rs10817314
SLC20A2	8p11.21	6575	Solute carrier family 20 member 2	0.0015	33	2	1	rs7845666
MAP4K3	2p22.1	8491	Mitogen-activated protein kinase kinase kinase kinase 3	0.0016	52	1	1	rs17508058
MGST3	1q24.1	4259	Microsomal glutathione S-transferase 3	0.0016	60	3	1	rs55977919
CLRN2	4p15.32	645,104	Clarin 2	0.0017	719	41	18	rs1498599
FOLH1	11q14.3	219,595	Folate hydrolase 1	0.0017	11	5	3	rs679470
GUCY1A3	4q32.1	2982	Guanylate cyclase 1 soluble subunit alpha	0.0018	77	1	1	rs62327005
UNC119B	12q24.31	84,747	Unc-119 lipid-binding chaperone B	0.0018	27	1	1	rs12825376

*Chr* = chromosome

### Pathway analysis

To interpret a gene list derived from gene-based analysis, functional enrichment analysis was performed using bioinformatics tool, DAVID, and results are shown in Table 4. Thirteen biological pathways were enriched with post-RT pain in breast cancer patients (FDR-adjusted *p* value < 0.05). These 13 biological pathways were then clustered into two groups: glucuronidation activity and olfactory receptor activity (enrichment score 4.60 and 3.41, respectively). These biological activities included xenobiotic and drug metabolism, ascorbate and aldarate metabolism, and olfactory signal transduction, suggesting their roles in underlying mechanisms of post-RT pain.

**Table 4:** Top pathways enriched in patients with post-RT pain in breast cancer patients from DAVID functional annotation module analysis

Cluster	Category	Term	No. genes in a term	Fold enrichment^1^	*p* value^2^	FDR^3^
1	GOTERM_BP_DIRECT	GO:0052697~xenobiotic glucuronidation	9	19.81952	3.45E-10	9.46E-07
	GOTERM_BP_DIRECT	GO:2001030~negative regulation of cellular glucuronidation	8	19.81952	6.14E-09	8.43E-06
	GOTERM_BP_DIRECT	GO:1904224~negative regulation of glucuronosyltransferase activity	8	19.81952	6.14E-09	8.43E-06
	GOTERM_BP_DIRECT	GO:0045922~negative regulation of fatty acid metabolic process	8	17.61735	2.64E-08	2.42E-05
	GOTERM_BP_DIRECT	GO:0052695~cellular glucuronidation	8	10.57041	3.62E-06	0.001983
	GOTERM_BP_DIRECT	GO:0052696~flavonoid glucuronidation	9	8.494079	4.53E-06	0.002071
	GOTERM_MF_DIRECT	GO:0015020~glucuronosyltransferase activity	9	7.057233	2.18E-05	0.019001
	GOTERM_MF_DIRECT	GO:0001972~retinoic acid binding	8	6.818583	1.04E-04	0.04456
	KEGG_PATHWAY	hsa00053:Ascorbate and aldarate metabolism	9	6.071802	6.39E-05	0.016351
	KEGG_PATHWAY	hsa00040:Pentose and glucuronate interconversions	10	5.159047	8.29E-05	0.007101
	KEGG_PATHWAY	hsa00860:Porphyrin and chlorophyll metabolism	11	4.706058	7.16E-05	0.009189
	KEGG_PATHWAY	hsa05204:Chemical carcinogenesis	13	3.081485	8.48E-04	0.053271
	KEGG_PATHWAY	hsa00982:Drug metabolism - cytochrome P450	12	3.189229	0.001097	0.055041
2	GOTERM_BP_DIRECT	GO:0050911~detection of chemical stimulus involved in sensory perception of smell	37	2.020171	8.21E-05	0.031689
	GOTERM_MF_DIRECT	GO:0004984~olfactory receptor activity	37	1.992656	1.09E-04	0.03161
	KEGG_PATHWAY	hsa04740:Olfactory transduction	36	1.730048	0.001446	0.060346
	GOTERM_MF_DIRECT	GO:0004930~G-protein coupled receptor activity	48	1.589467	0.0017	0.312315

^1^The fold enrichment is defined as the ratio of the two proportions; one is the proportion of genes in your list belong to certain pathway, and the other is the proportion of genes in the background information (i.e., universe genes) that belong to that pathway

^2^*p* values from modified Fisher’s exact test

^3^*FDR*, false discovery rate from Benjamini and Hochberg

*DAVID* = Database for Annotation, Visualization and Integrated Discovery, *GO* = Gene Ontology, *KEGG* = The Kyoto Encyclopedia of Genes and Genomes

## Discussion

This study reported results of the first GWAS of acute post-RT pain in breast cancer patients who had undergone adjuvant RT after surgery. Although no individual association reached genome-wide significance, collectively our results suggest genetic involvement in acute post-RT pain. These results, like all large-scale agnostic search for genetic associations, need validation. At the completion of RT, about 29% of patients reported having clinically relevant pain; of this subset, 30% reported moderate or severe levels of pain at pre-RT, while 70% had no or mild pain at pre-RT. The most significant factor associated with post-RT pain was the presence of pre-RT pain, which is in line with literature reporting that prior pain is the most significant prognostic factor for pain persistence [8, 25]. Besides pre-RT pain, other potential risk factors identified from multivariable regression analyses were included as covariates in the subsequent genetic association analyses to control for confounding effects. We conducted gene-based association analyses and functional enrichment analyses to identify additional loci complementary to GWAS. We identified four suggestive susceptibility loci from GWAS, seven suggestive genes from gene-level analysis, and two significantly enriched functional pathways associated with post-RT pain.

First, we reported four suggestive susceptibility loci for post-RT pain, rs16970540 (17q12), rs4584690 (13q32.1), rs7335912 (13q32.1), and rs73633565 (Xp22.2) proximal to three genes. The most significant marker, rs16970540, is mapped to the 3′-untranslated region (UTR) of *RFFL* gene or close to *LIG3* in chromosome 17. *RFFL* encodes a protein that regulates several biological processes through the ubiquitin-mediated proteasomal degradation of various target proteins. In the context of irradiation, *RFFL* negatively regulates p53/tumor protein 53 (TP53), the expression of which can be activated by radiation, directly, or indirectly through its ubiquitination [26]. The loss of TP53 function was related to sensitivity to ionizing radiation. The fraction of p53-positive fibroblasts was significantly higher in cultures from RT-sensitive patients compared to RT-resistant patients after in vitro irradiation [27]. Thus, *RFFL* can mediate radiation sensitivity via regulation of TP53. On the other hand, *LIG3* encodes a protein that catalyzes the joining of DNA ends and is involved in DNA replication, recombination, and repair. *LIG3* corrects defective DNA strand-break repair and sister chromatid exchange following RT through base excision repair and alternative non-homologous end-joining pathways. Polymorphisms near the *LIG3* gene (rs3744355, rs2074518, and rs3744357) have been reported to be associated with acute breast skin toxicity following RT both in a Japanese cohort (*n* = 399) and a European Caucasian cohort (*n* = 480) [28, 29]. It is possible that acute skin toxicity may lead to acute post-RT pain [30]. Thus, *LIG3* gene may not be specific to pain, and they can rather be applied to a more common genetic susceptibility to acute RT-induced normal tissue toxicities.

The next significant markers, rs4584690 and rs7335912, were mapped to *ABCC4/MRP4* gene, and three additional signals from the list of top 30 SNPs were also mapped to this gene. The Manhattan plot shows a stack of points in chromosome 13 (Fig. 1), which implies a possible haploblock structure and suggests a potential strong association of *ABCC4/MRP4* with post-RT pain. The range of pair-wise LD among five SNPs was 0.89–1.00 in CEU population according to the SNAP (https://data.broadinstitute.org/mpg/snpsnap/match_snps.html) (Additional File 2: Fig. S2). *ABCC4/MRP4* encodes a protein that is a member of ATP-binding cassette (ABC) transporter superfamily as well as a member of multidrug resistance-associated proteins (MRPs). *ABCC4/MPR4* transports most prostaglandins (PGs), which can sensitize spinal neurons to pain. In an animal study with mrp4-knockout mice, Lin et al. showed that a deficiency of mrp4 function led to a significant reduction of extracellular PG levels and consequent altered inflammatory nociceptive responses via modulating cAMP-mediated signaling pathway [31]. In a human candidate gene approach study, *ABCC4* rs9524885 has been associated with reduced pain among patients with non-small cell lung cancer [32].

Additionally, we searched gene regulation databases using HaploReg v4.1 (https://pubs.broadinstitute.org/mammals/haploreg/haploreg.php) to explore the potential roles of SNPs rs16970540, rs4584690, rs7335912, and rs73633565 as expression quantitative trait loci (eQTLs); rs16970540 exhibited direct eQTL effects (in total 19 hits) in regulating expressions of LIG3 in 12 tissues including blood, skin, nerve, and breast mammary tissues. According to GTEx Portal (https://www.gtexportal.org/home/), for instance, those who were heterozygous (CT) or homozygous (TT) for the minor allele of rs16970540 showed higher expression of LIG3 in breast tissue compared to those homozygous (CC) for the reference allele (OR = 2.63 per allele, *p* = 5.1 × 10^−8^).

In gene-based association analyses, we found seven susceptibility genes for post-RT pain: *EIF4G1, FAM131A, GRID2IP, NMUR2, OR10V1, CYP4F22*, and *LECT1*. This suggests the involvement of neurotransmitters, olfactory receptor genes, and cytochrome P450 in post-RT pain. Among these genes, Neuromedin U Receptor 2 (*NMUR2*) has been found to have a role in nociception and inflammation. *NMUR2* encodes a receptor protein for Neuromedin, which is a neuropeptide that is widely distributed in the central nervous system. Neuromedin U receptors are a group of Gq/11-protein-coupled receptors. In animal studies, *NMUR2*-null mice showed a reduced thermal nociceptive response in the hot plate tests and a significant reduction in acute chemo-nociception following capsaicin or formalin injection [33], by inhibiting T-type Ca2+ channel currents via pertussis toxin-sensitive protein kinase A pathway in a dose-dependent manner in mouse small dorsal root ganglion neurons [34]. However, one recent study reported that *NMUR2* did not play a role in the development of mechanical hypersensitivity following nerve injury by showing that there were no significant differences in heat hyperalgesia between wild-type and NMUR2-null mice [35]. Further studies are needed to confirm the involvement of *NMUR2* in mechanical hypersensitivity in humans, including patients with cancer.

To date, several genome-wide association studies of pain have been reported. According to the NHGRI-EBI GWAS Catalog [36, 37], a total of eight studies reported 30 SNPs associated with any pain. Among these eight studies, four studies reported nine SNPs reaching a genome-wide significance (*p* values ≤ 5 × 10^−8^) [38–41], while the other four studies identified suggestive susceptibility loci with *p* values ≤ 5 × 10^−6^ [42–45]. In 2013, Kim et al. reported the first GWAS of pain that identified rs2562456 in *ZNF429* gene to be significantly associated with acute post-surgery pain (*N* = 112; *p* = 2.0 × 10^−10^) [38]. In 2013, a large study with 7099 Europeans reported another genome-wide significant SNP, rs13361160 in *CCT5* gene for widespread pain (*p* = 1.2 × 10^−8^). However, this significance was attenuated when it was combined with the replication sample of 9469 Europeans (*p* = 4.7 × 10^−7^) [44].

In 2016, Reyes-Gibby et al. reported another genome-wide significant SNP, rs3862188 in *RP11-634B7.4* gene for severe pre-treatment pain in head and neck cancer patients (*N* = 1368; *p* = 3 × 10^−8^) [39]. Reyes-Gibby et al. identified 2 years later additional four SNPs which had statistically significant associations with neuropathic pain in head and neck cancer patients (*N* = 1043; rs10950641, *p* = 3 × 10^−14^; rs4804217, *p* = 3 × 10^−9^; rs6796803, *p* = 6 × 10^−9^; rs4775319, *p* = 1 × 10^−8^) [40]. These findings suggest that statistical power may increase when GWAS targets specific types of pain such as neuropathic pain. Another approach to increase statistical power would be a meta-analysis. A recent study of meta-analysis of individual data from 15 cohorts (*N* = 158,000) reported three SNPs significantly associated with chronic back pain (rs12310519, *p* = 5 × 10^−19^; rs7833174, *p* = 4 × 10^−13^; rs4384683, *p* = 2 × 10^−10^) [41]. Susceptibility genes identified previously [36, 37] for any type of pain traits included *CD3E*, *HMGB1P46, C5, DDC, DIS3L2, ESRRG, GFRA2, DOK2, GPD2, IL1R1, LCLAT1, MCM3, PRKCA, RORA, SNX8, SOX5, TESC,* and *ZSCAN20* [36]. None of these genes reached the pre-defined significance level of *p* < 2.5 × 10^−6^ in our study.

We identified 13 enriched biological pathways for post-RT pain, which were clustered into two groups by DAVID functional annotation module: glucuronidation and olfactory receptor activities. Glucuronidation activity is involved in detoxification and xenobiotic metabolism of substances such as drugs, bilirubin, and fatty-acid derivatives. Glucuronidation transfers glucuronic acid component of uridine diphosphate (UDP)-glucuronic acid to a substrate by UDP-glucuronosyltransferase to make substances more water-soluble, so they can be excreted from body or less toxic. The ascorbate and aldarate metabolism pathway include glucuronidation in the upstream processes of ascorbate synthesis. Ascorbate, which is well known as vitamin C, plays a critical role as an antioxidant in many biological processes such as detoxification of exogenous compounds. Vitamin C has a beneficial effect on pain relief in different pain conditions including cancer pain by decreasing oxidative stress and/or inflammation, which can both be caused by anti-cancer treatments [46, 47]. Ascorbate also functions as a cofactor for a family of enzymes involved in the biosynthesis of neurotransmitters and neuropeptide hormones that can modulate pain transmission.

Olfactory receptor activity can be aligned with our findings of *OR10V1* as one of the genes associated with post-RT pain. We also found three additional olfactory receptor genes (*OR52N1, OR4C12, OR4A47*) included in the top 30 genes. Recently, Reyes-Gibby et al. have reported that genetic variants in RP11-634B7.4 gene, which is annotated as antisense to the three olfactory receptor genes, *OR13G1, OR6F1*, and *OR14A2*, were significantly associated with severe pre-treatment pain among patients with head and neck cancer at genome-wide significance levels [39]. The olfactory receptors are members of G-protein-coupled receptors, which are involved in signal transduction and play important roles in many physiological processes including sensory perception, regulation of behavior and mood, regulation of immune system activity and inflammation, and tumor growth and metastasis. The authors speculated that olfactory receptor genes may be involved in pain pathway via activating downstream mitogen-activated kinases (MAPK) signaling pathway [48], by linking to their previous finding of *MAPK1/ERK2* as a novel target gene for cancer pain [49]. In fact, there have been many animal experiments to modulate neuropathic cancer pain by inhibiting MAPK signaling pathway using upstream effectors, such as R419, adenosine monophosphate-activated protein kinase activator [50], and bisphosphonates [51]. Considering that majority of breast cancer pain is neuropathic in nature [52, 53], the investigation of a functional mechanism which connects olfactory receptors, MAPK pathway, and pain perception in breast cancer patients may seem worthwhile. More studies in larger populations are needed to validate our findings.

This study has several strengths and limitations. To the best of our knowledge, this is the first report of GWAS of post-RT pain among breast cancer patients of different race and ethnicity. Considering that majority of GWAS data currently available are for NHW, the results from diverse race/ethnic background have more potential for generalizability. Second, the ascertainment of outcome variables was relatively homogeneous compared to large consortium-based studies because we obtained self-reported pain severity data using the same questionnaires from all participating centers. The first limitation of this study is the relatively small sample size, which might have limited the statistical power of the analysis. Based on our findings of rs16970540, with minor allele frequency of 0.1, OR of 2.2, and type 1 error rate of 5 × 10^−8^, we had only 17% statistical power to be able to reject the null hypothesis. We will need 694 cases and 1673 controls to have at least 80% of statistical power. So, a larger joint GWAS with multiple cohorts is warranted to validate our findings. In addition to limited statistical power, the failure of GWAS may be attributed to the complex nature of the phenotype, post-RT pain, we evaluated. Pain is a more complex functional end-point, which is affected by multiple genes within a pathway rather than a simple Mendelian disease. We employed gene-based association analyses and pathway-based analyses to increase statistical power as well as to find additional genetic loci underlying molecular mechanisms of post-RT pain. Another limitation would be the lack of replication with an independent dataset.

## Conclusion

In the current study, we conducted GWAS, gene-based association analyses, and pathway-based functional enrichment analyses to evaluate the genetic risk loci for acute post-RT pain among breast cancer patients. We identified two biological processes, glucuronidation activity and olfactory receptor activity, in addition to the potential role of *LIG3*, *ABCC4/MPR4*, and *EGFL6* from GWAS, were involved in post-RT pain, which showed that post-RT pain is a polygenic trait. Post-RT pain can be affected by DNA damage/repair, transporter and receptor activity in signal transduction, and cellular detoxification via glucuronidation activity. Larger studies are warranted to validate our findings to facilitate the discovery of underlying genetic/molecular mechanisms of pain related to cancer treatments. The results can ultimately contribute to the development of prevention and/or intervention strategies to improve cancer pain management and QOL in cancer patients.

## Additional files

Additional file 1: **Figure S1.** Q-Q plots for post-RT pain. This figure shows the quantile-quantile plots of observed versus expected *p* values on the −log10 scale, showing the conformity of the observed results to expectations under the null. Black lines indicate the distribution of observed *p* value versus expected p value, and red lines indicate the null distribution. Lambda confirms appropriate control of population substructure; (a) 1.649 before adjustment, (b) 1.017 after adjusting for population substructure with the first 3 PCs, and (c) 1.016 after further adjusting for all potential confounders identified in Table 1. PCs: principal components. (DOCX 32 kb)

Additional file 2: **Figure S2.** Regional association plot for rs4584690 on chromosome 13 located nearby ABCC4/MRP4 gene. The *y* axis is −log10 of *p* values and *x* axis is the genomic location of each SNP. Linkage disequilibrium coefficients were derived from hg19 (1000 Genomes March 2012, European population) and local estimates of recombination rates are from HapMap samples (2008–03_rel22_B36; ftp://ftp.ncbi.nlm.nih.gov/hapmap/). The plot was generated using LocusZoom (http://locuszoom.org/). (DOCX 123 kb)

## Data Availability

The datasets used and/or analyzed during the current study are available from the corresponding author on reasonable request.

## Abbreviations

AAn: African American/black
BCS: Breast-conserving surgery
BMI: Body mass index
CI: Confidence interval
DAVID: Database for Annotation, Visualization and Integrated Discovery
eQTLs: Expression quantitative trait loci
GO: Gene Ontology
GWAS: Genome-wide association study
HER2: Human epidermal growth factor receptor 2
HW: Hispanic Whites
HWE: Hardy-Weinberg equilibrium
KEGG: Kyoto Encyclopedia of Genes and Genomes
LD: Linkage disequilibrium
NHW: Non-Hispanic Whites
OR: Odds ratio
PC: Principal component
PCA: Principal component analysis
QOL: Quality of life
Q-Q: Quantile-quantile
RT: Radiotherapy
SD: Standard deviation
SNP: Single-nucleotide polymorphism
UDP: Uridine diphosphate

## References

[1] Siegel RL, Miller KD, Jemal A (2018). Cancer statistics, 2018. CA Cancer J Clin.

[2] Miller KD, Siegel RL, Lin CC, Mariotto AB, Kramer JL, Rowland JH (2016). Cancer treatment and survivorship statistics, 2016. CA Cancer J Clin.

[3] Darby S, McGale P, Correa C, Taylor C, Arriagada R, Early Breast Cancer Trialists' Collaborative G (2011). Effect of radiotherapy after breast-conserving surgery on 10-year recurrence and 15-year breast cancer death: meta-analysis of individual patient data for 10,801 women in 17 randomised trials. Lancet..

[4] van den Beuken-van Everdingen MH, de Rijke JM, Kessels AG, Schouten HC, van Kleef M, Patijn J (2007). Prevalence of pain in patients with cancer: a systematic review of the past 40 years. Ann Oncol.

[5] Reyes-Gibby C, Morrow PK, Bennett MI, Jensen MP, Shete S (2010). Neuropathic pain in breast cancer survivors: using the ID pain as a screening tool. J Pain Symptom Manag.

[6] Lundstedt D, Gustafsson M, Malmstrom P, Johansson KA, Alsadius D, Sundberg A (2010). Symptoms 10-17 years after breast cancer radiotherapy data from the randomised SWEBCG91-RT trial. Radiother Oncol.

[7] Lundstedt D, Gustafsson M, Steineck G, Malmstrom P, Alsadius D, Sundberg A (2012). Risk factors of developing long-lasting breast pain after breast cancer radiotherapy. Int J Radiat Oncol Biol Phys.

[8] Lee E, Takita C, Wright JL, Reis IM, Zhao W, Nelson OL (2016). Characterization of risk factors for adjuvant radiotherapy-associated pain in a tri-racial/ethnic breast cancer population. Pain..

[9] McCann B, Miaskowski C, Koetters T, Baggott C, West C, Levine JD (2012). Associations between pro- and anti-inflammatory cytokine genes and breast pain in women prior to breast cancer surgery. J Pain.

[10] Stephens K, Cooper BA, West C, Paul SM, Baggott CR, Merriman JD (2014). Associations between cytokine gene variations and severe persistent breast pain in women following breast cancer surgery. J Pain.

[11] Caronia D, Martin M, Sastre J, de la Torre J, Garcia-Saenz JA, Alonso MR (2011). A polymorphism in the cytidine deaminase promoter predicts severe capecitabine-induced hand-foot syndrome. Clin Cancer Res.

[12] Garcia-Giralt N, Rodriguez-Sanz M, Prieto-Alhambra D, Servitja S, Torres-Del Pliego E, Balcells S (2013). Genetic determinants of aromatase inhibitor-related arthralgia: the B-ABLE cohort study. Breast Cancer Res Treat.

[13] Hu JJ, Urbanic JJ, Case LD, Takita C, Wright JL, Brown DR (2018). Association between inflammatory biomarker C-reactive protein and radiotherapy-induced early adverse skin reactions in a multiracial/ethnic breast cancer population. J Clin Oncol.

[14] Wright JL, Takita C, Reis IM, Zhao W, Lee E, Nelson OL (2016). Prospective evaluation of radiation-induced skin toxicity in a race/ethnically diverse breast cancer population. Cancer Med.

[15] Cleeland CS, Ryan KM (1994). Pain assessment: global use of the brief pain inventory. Ann Acad Med Singap.

[16] Butt Z, Wagner LI, Beaumont JL, Paice JA, Peterman AH, Shevrin D (2008). Use of a single-item screening tool to detect clinically significant fatigue, pain, distress, and anorexia in ambulatory cancer practice. J Pain Symptom Manag.

[17] Oldenmenger WH, de Raaf PJ, de Klerk C, van der Rijt CC (2013). Cut points on 0-10 numeric rating scales for symptoms included in the Edmonton symptom assessment scale in cancer patients: a systematic review. J Pain Symptom Manag.

[18] Purcell S, Neale B, Todd-Brown K, Thomas L, Ferreira MA, Bender D (2007). PLINK: a tool set for whole-genome association and population-based linkage analyses. Am J Hum Genet.

[19] Price AL, Patterson NJ, Plenge RM, Weinblatt ME, Shadick NA, Reich D (2006). Principal components analysis corrects for stratification in genome-wide association studies. Nat Genet.

[20] Dupont WD, Plummer WD (1990). Power and sample size calculations: a review and computer program. Control Clin Trials.

[21] Turner S. Ensembl/Entrez hg19/GRCh37 Consensus Genes: figshare; 2012 [Available from: https://figshare.com/articles/hg19_GRCh37_Consensus_Genes/103113/4. Accessed 3 Sep 2016

[22] Veyrieras JB, Kudaravalli S, Kim SY, Dermitzakis ET, Gilad Y, Stephens M (2008). High-resolution mapping of expression-QTLs yields insight into human gene regulation. PLoS Genet.

[23] Holmans P (2010). Statistical methods for pathway analysis of genome-wide data for association with complex genetic traits. Adv Genet.

[24] Huang d W, Sherman BT, Lempicki RA (2009). Systematic and integrative analysis of large gene lists using DAVID bioinformatics resources. Nat Protoc.

[25] Poleshuck EL, Katz J, Andrus CH, Hogan LA, Jung BF, Kulick DI (2006). Risk factors for chronic pain following breast cancer surgery: a prospective study. J Pain.

[26] Dong Y, Zhao J, Wu CW, Zhang L, Liu X, Kang W (2013). Tumor suppressor functions of miR-133a in colorectal cancer. Mol Cancer Res.

[27] Nuta O, Somaiah N, Boyle S, Chua ML, Gothard L, Yarnold J (2016). Correlation between the radiation responses of fibroblasts cultured from individual patients and the risk of late reaction after breast radiotherapy. Cancer Lett.

[28] Suga T, Ishikawa A, Kohda M, Otsuka Y, Yamada S, Yamamoto N (2007). Haplotype-based analysis of genes associated with risk of adverse skin reactions after radiotherapy in breast cancer patients. Int J Radiat Oncol Biol Phys.

[29] Murray RJ, Tanteles GA, Mills J, Perry A, Peat I, Osman A (2011). Association between single nucleotide polymorphisms in the DNA repair gene LIG3 and acute adverse skin reactions following radiotherapy. Radiother Oncol.

[30] Pignol J-P, Thuc Vu TT, Mitera G, Bosnic S, Verkooijen HM, Truong P (2015). Prospective evaluation of severe skin toxicity and pain during postmastectomy radiation therapy. Int J Radiat Oncol Biol Phys.

[31] Lin ZP, Zhu YL, Johnson DR, Rice KP, Nottoli T, Hains BC (2008). Disruption of cAMP and prostaglandin E2 transport by multidrug resistance protein 4 deficiency alters cAMP-mediated signaling and nociceptive response. Mol Pharmacol.

[32] Sloan JA, de Andrade M, Decker P, Wampfler J, Oswold C, Clark M (2012). Genetic variations and patient-reported quality of life among patients with lung cancer. J Clin Oncol.

[33] Torres R, Croll SD, Vercollone J, Reinhardt J, Griffiths J, Zabski S (2007). Mice genetically deficient in neuromedin U receptor 2, but not neuromedin U receptor 1, have impaired nociceptive responses. Pain..

[34] Wang F, Zhang Y, Jiang X, Zhang Y, Zhang L, Gong S (2011). Neuromedin U inhibits T-type Ca2+ channel currents and decreases membrane excitability in small dorsal root ganglia neurons in mice. Cell Calcium.

[35] Gilbert AK, Puma C, Xu X, Laird JM (2013). Neuromedin U receptor 2 does not play a role in the development of neuropathic pain following nerve injury in mice. Eur J Pain (London, England)..

[36] MacArthur J, Bowler E, Cerezo M, Gil L, Hall P, Hastings E (2017). The new NHGRI-EBI catalog of published genome-wide association studies (GWAS catalog). Nucleic Acids Res.

[37] Buniello A, MacArthur JAL, Cerezo M, Harris LW, Hayhurst J, Malangone C (2019). The NHGRI-EBI GWAS catalog of published genome-wide association studies, targeted arrays and summary statistics 2019. Nucleic Acids Res.

[38] Kim H, Ramsay E, Lee H, Wahl S, Dionne RA (2009). Genome-wide association study of acute post-surgical pain in humans. Pharmacogenomics..

[39] Reyes-Gibby CC, Wang J, Silvas MR, Yu RK, Hanna EY, Shete S (2016). Genome-wide association study suggests common variants within RP11-634B7.4 gene influencing severe pre-treatment pain in head and neck cancer patients. Sci Rep.

[40] Reyes-Gibby CC, Wang J, Yeung SJ, Chaftari P, Yu RK, Hanna EY (2018). Genome-wide association study identifies genes associated with neuropathy in patients with head and neck cancer. Sci Rep.

[41] Suri P, Palmer MR, Tsepilov YA, Freidin MB, Boer CG, Yau MS (2018). Genome-wide meta-analysis of 158,000 individuals of European ancestry identifies three loci associated with chronic back pain. PLoS Genet.

[42] Meng W, Deshmukh HA, van Zuydam NR, Liu Y, Donnelly LA, Zhou K (2015). A genome-wide association study suggests an association of Chr8p21.3 (GFRA2) with diabetic neuropathic pain. Eur J Pain (London, England).

[43] Meng W, Deshmukh HA, Donnelly LA (2015). Wellcome Trust Case control C, surrogate markers for M, macro-vascular hard endpoints for innovative diabetes tools study g, et al. a genome-wide association study provides evidence of sex-specific involvement of Chr1p35.1 (ZSCAN20-TLR12P) and Chr8p23.1 (HMGB1P46) with diabetic neuropathic pain. EBioMedicine..

[44] Peters MJ, Broer L, Willemen HL, Eiriksdottir G, Hocking LJ, Holliday KL (2013). Genome-wide association study meta-analysis of chronic widespread pain: evidence for involvement of the 5p15.2 region. Ann Rheum Dis.

[45] Warner Sophie C, van Meurs Joyce BJ, Schiphof Dieuwke, Bierma-Zeinstra Sita M, Hofman Albert, Uitterlinden Andre G, Richardson Helen, Jenkins Wendy, Doherty Michael, Valdes Ana M (2017). Genome-wide association scan of neuropathic pain symptoms post total joint replacement highlights a variant in the protein-kinase C gene. European Journal of Human Genetics.

[46] Gunes-Bayir A, Kiziltan HS (2015). Palliative vitamin C application in patients with radiotherapy-resistant bone metastases: a retrospective study. Nutr Cancer.

[47] Carr AC, Vissers MC, Cook JS (2014). The effect of intravenous vitamin C on cancer- and chemotherapy-related fatigue and quality of life. Front Oncol.

[48] Benbernou N, Esnault S, Galibert F (2013). Activation of SRE and AP1 by olfactory receptors via the MAPK and rho dependent pathways. Cell Signal.

[49] Reyes-Gibby CC, Wang J, Silvas MR, Yu R, Yeung SC, Shete S (2016). MAPK1/ERK2 as novel target genes for pain in head and neck cancer patients. BMC Genet.

[50] Mejia Galo L., Asiedu Marina N., Hitoshi Yasumichi, Dussor Gregory, Price Theodore J. (2016). The potent, indirect adenosine monophosphate-activated protein kinase activator R419 attenuates mitogen-activated protein kinase signaling, inhibits nociceptor excitability, and reduces pain hypersensitivity in mice. PAIN Reports.

[51] Yao Y, Tan YH, Light AR, Mao J, Yu AC, Fu KY (2016). Alendronate Attenuates Spinal microglial activation and neuropathic pain. J Pain.

[52] Bokhari FN, McMillan DE, McClement S, Daeninck PJ (2012). Pilot study of a survey to identify the prevalence of and risk factors for chronic neuropathic pain following breast cancer surgery. Oncol Nurs Forum.

[53] Gartner R, Jensen MB, Nielsen J, Ewertz M, Kroman N, Kehlet H (2009). Prevalence of and factors associated with persistent pain following breast cancer surgery. J Am Med Assoc.

