# Supplementary figures and images for: Genome-wide enriched pathway analysis of acute post-radiotherapy pain in breast cancer patients: a prospective cohort study
Source: Hum Genomics. 2019 Jun 13;13:28. doi: 10.1186/s40246-019-0212-8 (PMC6567461; doi:10.1186/s40246-019-0212-8)

**
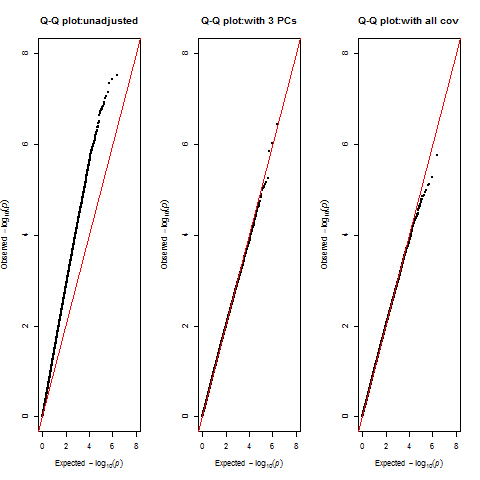
**

**Figure S1**

Supplement: Supplementary file 1 — Figure S1. Q-Q plots for post-RT pain. This figure shows the quantile-quantile plots of observed versus expected p values on the −log10 scale, showing the conformity of the observed results to expectations under the null. Black lines indicate the distribution of observed p value versus expected p value, and red lines indicate the null distribution. Lambda confirms appropriate control of population substructure; (a) 1.649 before adjustment, (b) 1.017 after adjusting for population substructure with the first 3 PCs, and (c) 1.016 after further adjusting for all potential confounders identified in Table 1. PCs: principal components. (DOCX 32 kb) [file 40246_2019_212_MOESM1_ESM.docx]
